# An open-source motion platform that replicates time synchronised internal and external patient motion for real-time image-guided radiotherapy

**DOI:** 10.1007/s13246-026-01710-w

**Published:** 2026-02-17

**Authors:** Alicja Kaczynska, Chris Kuban, Abbas Zahr, William Nixon, Paul Keall, Freeman Jin, Ann Yan, Daniel Mason, Maegan Stewart, Julia Johnson, Jonathan Hindmarsh, Chandrima Sengupta

**Affiliations:** 1 Image X Institute, Faculty of Medicine and Health, The University of Sydney, Sydney, Australia; 2 Nepean Cancer and Wellness Centre, Nepean Hospital, Sydney, Australia; 3 Northern Sydney Cancer Centre, Royal North Shore Hospital, Sydney, Australia

**Keywords:** Intrafraction motion, 6 Degree-of-freedom quality assurance, Motion platform, Robotic motion phantom, Real-time image guided radiotherapy

## Abstract

Real-time Image-guided Radiotherapy (IGRT) technologies aim to track intra-fractional tumour motion during delivery of high radiation doses to tumours. For the development and safe implementation of real-time IGRT technologies into the clinic, there is a need for robust and repeatable quality assurance (QA) devices. Motivated by this need, this work presents the development and characterisation of a novel time-synchronised motion platform designed for QA purposes of real-time IGRT technologies that perform combined internal and external patient motion monitoring. The *Int*ernal-*E*xternal *R*obotic *Act*uator (IntERAct) QA device was developed to integrate a 6-degree-of-freedom (6DoF) robotic arm with a 1-degree-of-freedom (1DoF) motion actuator, which replicate 6DoF internal tumour and 1DoF external surface movements, respectively. The IntERAct device was validated by performing tests which replicated patient-measured lung and liver motion traces on the 6DoF and 1DoF platforms. The device synchronised the internal and external motions to within 0.1 s with under two-millimetre geometric accuracy. The full details of the IntERAct device have been compiled into an open-source repository on GitHub for the medical physics community to use: https://github.com/Image-X-Institute/IntERAct.

## Introduction

A significant challenge in radiotherapy is achieving precise cancer targeting while sparing surrounding healthy tissues. This challenge occurs in part due to tumour motion caused by unavoidable physiological processes such as respiration. This motion can lead to clinically significant tumour underdosing and toxicities to organs at risk [1, 2]. Advanced motion management is essential for addressing the challenges of intrafraction variations in radiotherapy. Real-time image-guided radiotherapy (IGRT) solutions continuously monitor the tumour position, with potential to facilitate real-time treatment adaptation [2].

Real-time IGRT can be performed on dedicated radiation therapy devices including the CyberKnife robotic radiosurgery system [3], the Radixact helical TomoTherapy system [4], the ExacTrac system [5], and MRI-Linacs [6]. Several of these systems monitor tumour motion using a combination of occasional internal x-ray imaging and continuous optical/infrared surface imaging. This combination of external, non-ionising motion monitoring (often used for tumour sites which move with respiration) with more accurate but non-continuous x-ray imaging enables reduced patient imaging dose relative to continuous x-ray imaging [2]. The CyberKnife system generates a correlation model between the location of tumours on kilovoltage x-ray images with the motion of external LED markers on the patient’s body [7]. The Radixact system similarly uses a correlation model between internal target positions on x-ray images with LED marker positions on the patient surface [8]. Combined internal and external imaging for liver tumour motion monitoring has also been performed on a standard linear accelerator using the Combined Optical and Sparse Monoscopic Imaging with Kilovoltage x-rays (COSMIK) IGRT technology [9, 10]. A six degrees-of-freedom internal-external correlation model for translational and rotational tumour motion monitoring has also been investigated using data from a lung Stereotactic Ablative Body Radiotherapy (SABR) patient cohort [11].

To implement a comprehensive real-time IGRT tracking technology safely in the clinic, a specialised quality assurance (QA) device that can replicate known, real-time motion to evaluate the performance of the tracking technology is needed. A key requirement of a motion platform for quality assurance in research and clinical applications is that the system can replicate the full range of observed patient motion [12, 13]. For quality assurance and experimental validation of real-time IGRT systems combining internal target and external surface motion through a correlation modelling framework, such motion platforms need to combine time-synchronised 6DoF internal and 1DoF external motion components.

Existing commercially available QA devices, like the HexaMotion platform (ScandiDos AB, Uppsala, Sweden), have limitations in their motion range and cost-effectiveness. While the HexaMotion platform demonstrates a favourable dynamic localisation accuracy of ≤ 0.3 mm [14], it is restricted to motion in five degrees-of-freedom (DoF) with limited rotation capabilities of ± 10° roll motion, + 3/−6° tilt motion, and no yaw rotation. Tumours in the lung can rotate up to 45° [15] and liver tumours can rotate up to 29° [16] during treatment; for comprehensive validation these QA devices should be able to reproduce such movements. HexaMotion is also designed for specific use with the Delta4 phantom so is not as cost-effective as non-device specific systems [12]. Other existing commercial phantoms like QUASAR pRESP and CIRS can typically replicate only 3DoF translational tumour movement, or 3DoF translation plus 1DoF rotation only, with some systems also integrating 1DoF external motion replication [13].

Recent studies have introduced dynamic motion platforms to replicate internal and external patient tumour motion. However, these motion platforms are often limited in that the full extent of 6DoF internal tumour motion cannot be reproduced. A dynamic motion platform with two drive systems to enable 3DoF tumour and synchronised 3DoF external surface motion replication achieved positional accuracy < 0.2 mm [13]. Additionally, an externally deformable thoracic motion phantom was developed to simulate 1DoF tumour and whole-surface motion by driving a simulated diaphragm within a modified anthropomorphic chest phantom [17]. While the platform can replicate whole-surface external motion, it is driven by a purely 1DoF motion actuator so is unable to replicate 6DoF internal tumour positions from actual patients.

This work describes the development and experimental implementation of an *Int*ernal-*E*xternal *R*obotic *Act*uator (IntERAct), an open-source quality assurance device designed to replicate the full extent of 6DoF internal tumour motion and 1DoF external motion for the purpose of testing combined internal-external or multi-target motion monitoring technologies. The purpose of this study was to develop and characterise the performance of the IntERAct QA device in a laboratory environment and clinical setting. We aimed to develop the IntERAct device to be able to achieve repeatable millimetric accuracy and time synchronisation between the internal 6DoF and external 1DoF motion platform components of < 0.1 s.

## Methods

**Fig. 1:**
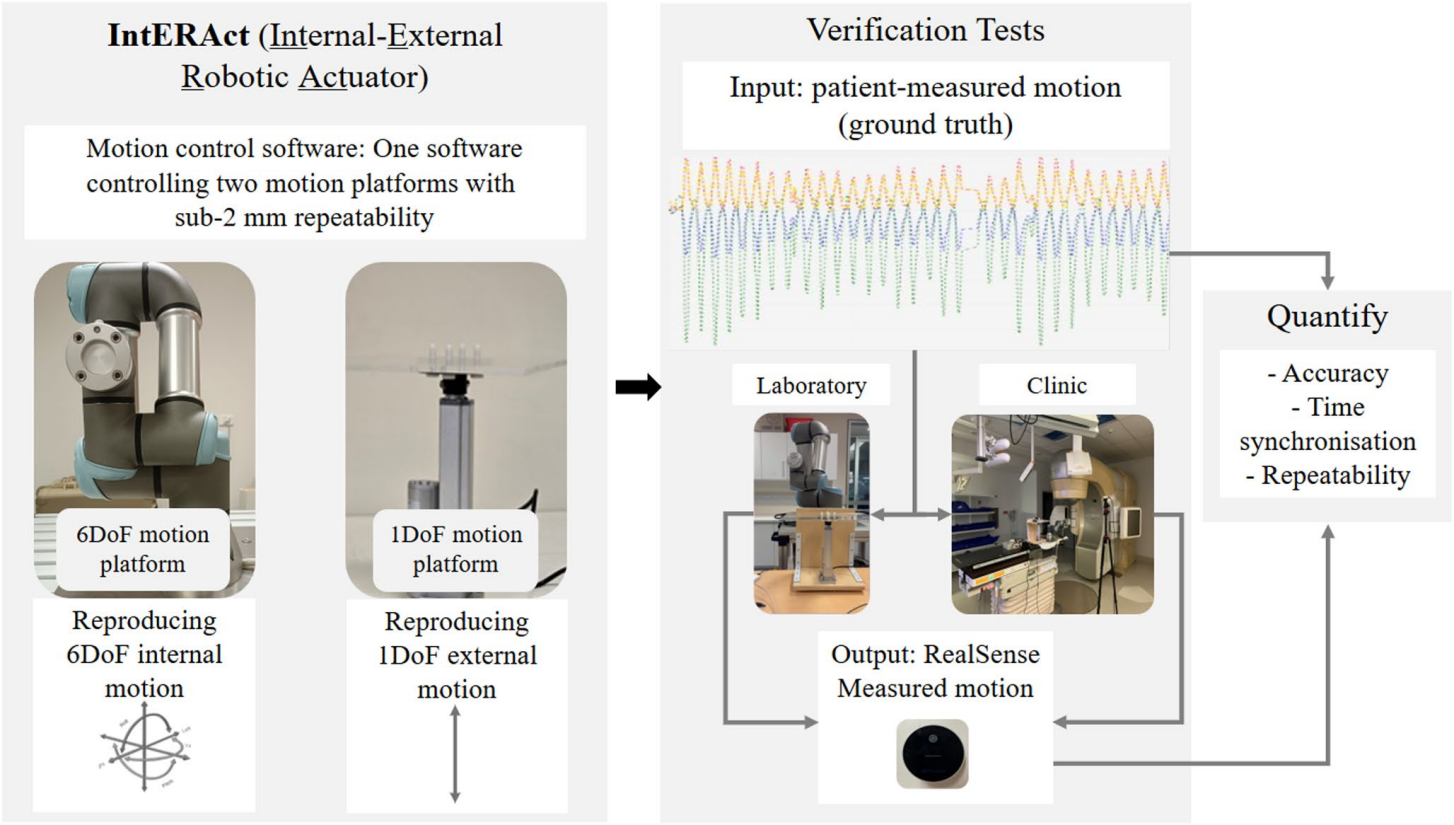
Overview of the IntERAct quality assurance device and verification tests conducted in this work. IntERAct consists of a motion control software controlling 6DoF and 1DoF motion platforms to reproduce internal and external patient motion, respectively. IntERAct was used to actuate patient-measured motion traces in the laboratory and the clinic, with motion measured by a RealSense depth-sensing camera. Geometric and time synchronisation accuracy and repeatability were measured to characterise the performance

The IntERAct QA device comprises of three components: a 6DoF motion platform programmed to replicate 6DoF internal tumour motion, a 1DoF platform programmed to replicate the dominant 1DoF respiratory component of patient surface motion (anterior-posterior motion), and an open-source motion control software that controls both platforms (Fig. 1).

### 6DoF motion platform

**Fig. 2:**
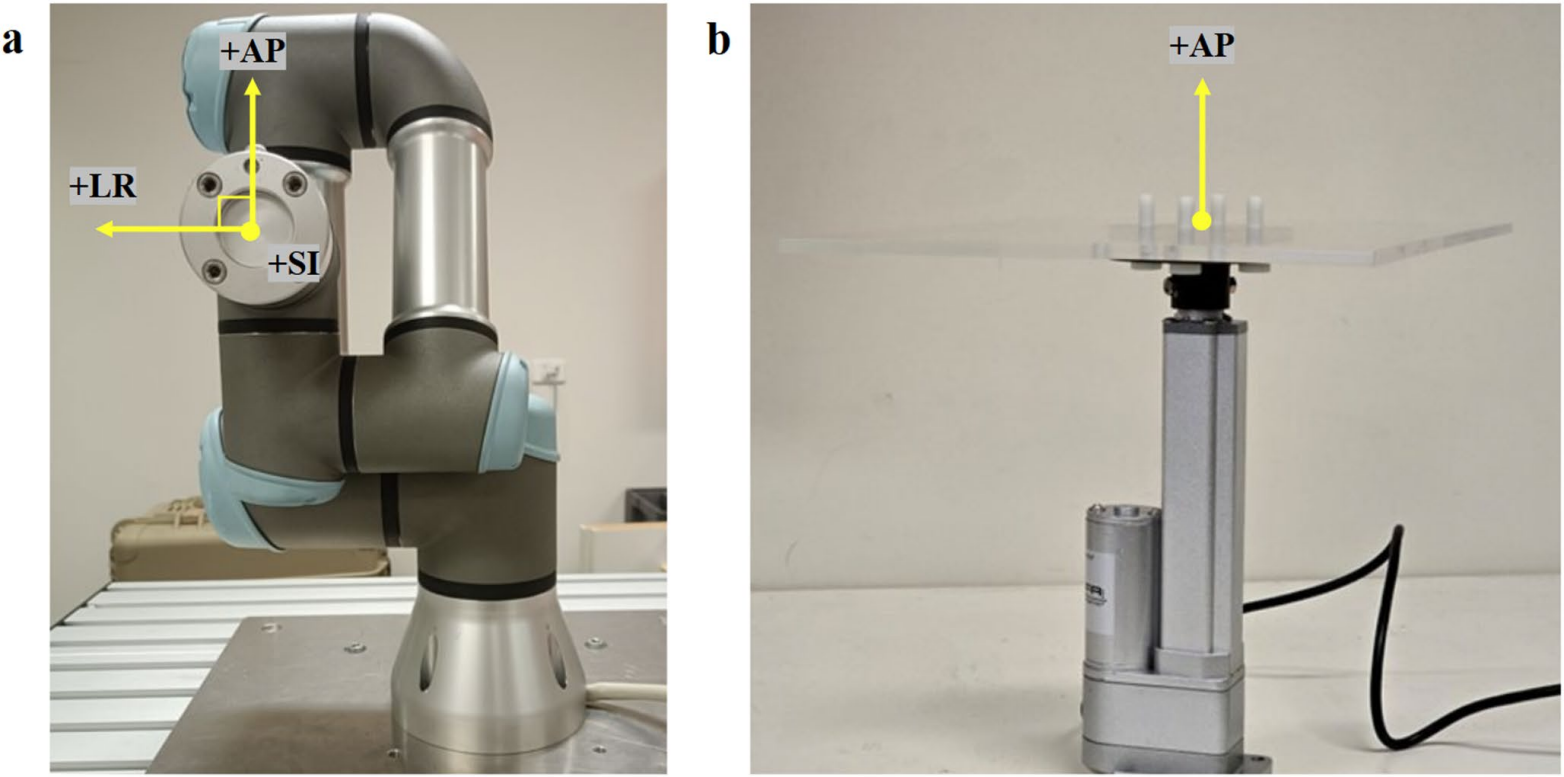
**a** Setup of the 6DoF motion platform component with coordinate system following IEC 61217 convention. **b** Setup of the 1DoF platform component with coordinate system following IEC 61217 convention

The 6DoF motion platform (Fig. 2a) is a commercially available robotic arm from Universal Robots (Odense, Denmark) which can be used to replicate 3DoF translational and 3DoF rotational components of internal tumour motion using a motion control software described in Section “IntERAct motion control software”. A QA device was initially developed [18] for the UR3 robotic arm design [19] and has been upgraded to also operate with the UR16 design [20]. The robotic arms from Universal Robots were chosen for this application due to their position repeatability (± 0.1 mm) and large angular range (± 360° for all axes). This system is fully programmable and supports up to 16 kg payload (UR16) at a reach of approximately 900 mm. This motion range allows attached phantoms to be extended into the treatment field without having any mechanical support below the phantom that may impact image quality during tracking. Phantoms that can be attached include those embedded with tracking gold markers or Calypso (Varian) electromagnetic transponders, or a marker-less phantom [21] and a dosimeter [22] to accurately replicate tumour motion patterns observed in real patients.

The UR robot must be set up with adequate clearance between the robot flange and the linac head, accounting for the programmed motion range and expected deviations from the setup position. We recommend a setup margin of at least 5 cm beyond the programmed motion, considering the rotation of the linac gantry. Safety limits enforcing the maximum extension from the robot centre position are configurable using the UR robot controller and can be adjusted to setup requirements [23]. Safety limits are also implemented in the IntERAct motion control software to assist in preventing collisions. The robot movement is immediately stopped if the measured motion from the robot feedback system exceeds the maximum motion range in the input motion trace by ± 5 mm in any of the three translational directions.

### 1DoF motion platform

The main component of the 1DoF component platform is a linear actuator from Firgelli Automation (Ferndale, WA, United States) [24] which converts rotational motion into linear motion using DC motors. The actuator can push or pull with a maximum load of 15.9 kg. The actuator can fully extend up to 101 mm which is sufficient to cover the maximum range of respiratory motions observed during radiotherapy [1]. The input voltage is 12 V DC, with the platform being driven by a 12 V DC Motor Drive [25] (5 A) which is a module that connects to the actuator and an Arduino UNO microcontroller board (Arduino, Monza, Italy) [26] (5 V and 25 mA) to control its velocity. The fully retracted position refers to the 0 minimum value of the 1DoF platform (Fig. 2b).

To determine the relationship between the input voltage and the 1DoF platform’s velocity, the platform was moved for different voltage values for 1 s. An Intel RealSense L515 depth-sensing camera (Intel RealSense, Santa Clara, CA, United States) [27] was used to measure the displacement of the 1DoF platform given varying voltage values. Following these measurements, the Eq. (1) was obtained by using a linear fit:

1 $$\left\{\begin{array}{l}V=\frac{\left(v+2.5\right)}{0.2265}\,(v<0)\\ V=0\,\left(v=0\right)\\ V=\frac{(v+3.9)}{0.2229}\,(v>0)\end{array}\right.$$

with *V* the voltage and *v* the velocity of the 1DoF platform in mm/s (distance measurements/1 s). This voltage-velocity relationship was used across all experimental validation of the 1DoF motion platform, as the operation of the platform was identical across all use of the platform in the vertical configuration.

### IntERAct motion control software

**Fig. 3:**
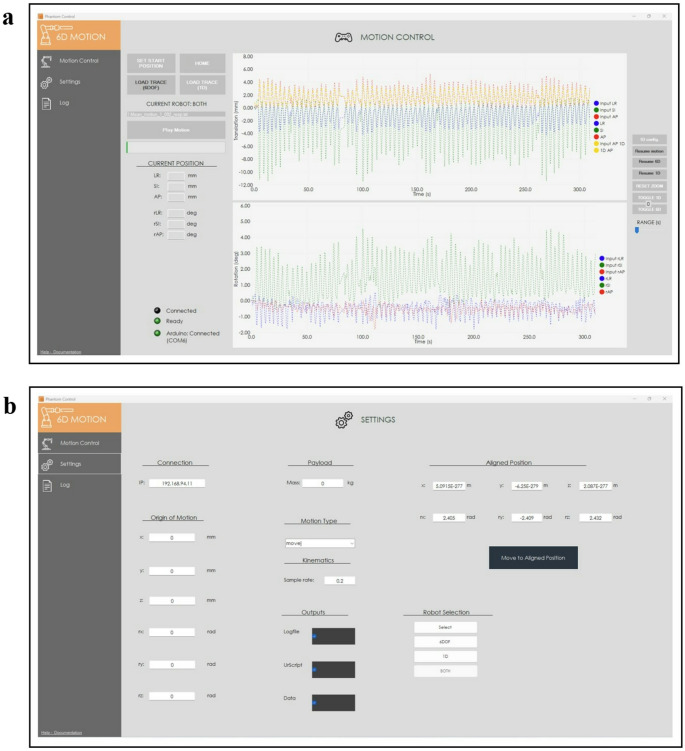
The IntERAct motion control software with **a** Motion Control tab with buttons to set the start position, buttons to load 6DoF and 1DoF motion traces and to play the motion and set and return the motion platform to the home position. **b** Settings page where the use of either the 6DoF or 1DoF platform alone or the entire combined 6DoF and 1DoF IntERAct QA device can be selected, 6DoF payload can be input, and temporal frequency can be selected

To drive the two motion platforms synchronously, the IntERAct motion control software was written in C# using Microsoft Visual Studio 2022 with .NET Framework 4.5, advanced from a previously developed software controlling the 6DoF motion platform [18]. The updated software controls both the 6DoF and 1DoF robotic motion platforms (Fig. 3). For the integration of 1DoF motion control, a C# code was written that sends three information packets: the voltage, a Boolean variable of the direction of each movement in the anterior-posterior direction, and the duration that the voltage should be applied. A Serial Port connection [28] enables communication between the IntERAct motion control software and the 1DoF platform, while the 6DoF motion platform can communicate with the motion control software through Modbus Transmission Control Protocol/Internet Protocol (TCP/IP). To synchronise the two platforms, the 6DoF motion platform’s real-time position feedback system (MODBUS register 400–405 [19, 20]) is used, with the motion control software waiting for the first 6DoF position feedback to be received, to then trigger 1DoF platform motion. A detailed software description and a step-by-step operational manual are available on our open-source repository [29].

To make the IntERAct QA device open-source, previous algorithms [18] were rewritten using open-source software packages, eliminating the need for external closed-source software packages. The updated software can synchronously run both motion platforms and has functionality for the UR16 robotic arm, allowing heavier phantom loads. New operational features of the motion control software include (1) a method to apply the ± 5 mm safety tolerance between programmed and actuated motion to prevent collisions with the linac, (2) a method to specify the treatment isocentre for easier device alignment, enabling automated QA setup, (3) a settings file to specify features of the input motion traces and phantom weight, and (4) a feature which writes log files of every action performed by the motion control software. The added software features allow accurate replication of the motion traces, the flexibility to run the motion traces as required, and the identification of any errors through the output log files.

### Performance characterisation of IntERAct

#### Validation tests

To validate the performance of the IntERAct QA device, QA tests were performed based on the American Association of Physicists in Medicine (AAPM) Task Group 40 (TG40) [30] and Task Group 142 (TG142) [31] protocols which recommend QA processes for radiation oncology and medical linear accelerators. The tests included dynamic localisation geometric accuracy, time synchronisation, and system repeatability. The recommended thresholds for medical linear accelerator QA spatial accuracy are 2 mm and 1° [30], however the requirements for SABR are stricter with spatial accuracy required to be < 1 mm and < 0.5° [31].

The performance of the IntERAct QA device was measured in a laboratory using patient-measured motion trace data, including measurements of the geometric accuracy and temporal synchronisation of the 6DoF and 1DoF platform components, and their repeatability. Following this laboratory testing, an experiment was performed in a radiotherapy clinic to test the QA device setup on the treatment couch and software features in a clinical setting. For these tests performed in the clinic, the geometric accuracy and repeatability of the QA device were measured.

#### Motion traces

The device is designed to be used for cancer sites that move with respiration. Data from this project were sourced from two clinical trials, LIGHT SABR [32] and LARK [33]. The trials measured intrafraction motion data for lung and liver cancer patients respectively, providing a broad range of patient-measured motion traces (Table 1). The LIGHT SABR trial measured 6DoF patient internal tumour motion using the Calypso electromagnetic transponder system, and a bellows belt collected chest expansion data used as a surrogate for patient external surface motion [32]. The LARK trial measured internal 6DoF patient internal tumour motion using the Kilovoltage Intrafraction Monitoring system based on kilovoltage x-ray imaging, and external 1DoF patient surface motion was collected using the Real-time Position Management system [33]. For the IntERAct QA device performance characterisation tests, the largest component of 6DoF internal motion (superior-inferior motion) alone was chosen to be actuated in the anterior-posterior direction by the 6DoF robotic arm, as a full characterisation of the 6DoF robotic arm had previously been performed [18]. The anterior-posterior motion of the patient surface was actuated in the anterior-posterior direction by the 1DoF motion platform.

The lower limits of normal diaphragmatic excursion during deep breathing have been indicated to be 32–42 mm from a study of 410 healthy volunteers [34]. A rapid diaphragmatic excursion could occur for instance when a patient coughs, which takes < 1 s. The presence of rapid diaphragmatic shifts in the patient data was quantified as the number of events in the 1DoF and 6DoF motion traces that correspond to a ≥ 10 mm motion range in ≤ 1 s. For the 6DoF motion traces, there were 126 events (36.6 s) in the Lung High Motion trace, 5 events (1.0 s) in the Liver Regular Motion 1 trace, and 50 events (14.6 s) in the Liver Regular Motion 2 trace. For the 1DoF motion traces, there were 24 events (3.8 s) in the Lung Regular Complexity trace and 99 events (57.4 s) in the Lung High Complexity trace. The input motion traces were 300 s long, so this indicates that rapid shifts were relatively infrequent across the entire cohort.

#### Measurements performed

A depth-sensing camera (Intel RealSense, Santa Clara, CA, United States) [27] was used to simultaneously measure anterior-posterior motion output by the two platforms. These measurements allowed the quantification of the mean $\bar{X}$, standard deviation $\sigma$, and 1st and 99th percentiles of the geometric differences between input and output motion traces as well as the time synchronisation between the two platforms. The purpose of measuring the anterior-posterior motion component alone for both the 1DoF and 6DoF platforms was to facilitate measurements of time synchronisation accuracy between the platforms and the 1DoF platform geometric accuracy, noting that the 6DoF platform’s geometric accuracy had already been verified in previous work [18]. For all seven traces tested in the laboratory, three repeatability tests have been conducted with the results presented in this table calculated by taking the average of all statistical quantifiers over the three tests. The standard deviation of these measurements gives an indication of the repeatability of the performance of the QA device.

**Table 1:** List of motion traces used for testing of the IntERAct quality assurance device, with motion characteristics

Clinical trial	Cancer site	Motion label	Motion platform	Mean ± standard deviation of motion range (mm)	Peak-to-peak motion range (mm)	Mean ± standard deviation of velocity (mm/s)	Maximum velocity (mm/s)
LIGHT SABR	Lung	Regular motion 1	6DoF	5.7 ± 1.6	7.9	0.0 ± 3.1	7.0
			1DoF	12.2 ± 9.5	55.1	0.0 ± 20.5	42.5
LIGHT SABR	Lung	Regular motion 2	6DoF	3.0 ± 1.9	6.4	0.0 ± 2.8	6.7
			1DoF	3.7 ± 2.6	12.9	0.0 ± 4.7	15.5
LIGHT SABR	Lung	High motion	6DoF	12.5 ± 5.1	21.0	0.0 ± 9.2	16.2
			1DoF	3.3 ± 2.2	9.7	0.0 ± 4.4	10.4
LIGHT SABR	Lung	Regular complexity	6DoF	6.3 ± 1.9	16.2	0.0 ± 2.4	11.4
			1DoF	3.8 ± 3.3	22.6	0.0 ± 6.2	21.0
LIGHT SABR	Lung	High complexity	6DoF	13.9 ± 2.9	18.5	0.0 ± 3.7	10.4
			1DoF	9.7 ± 6.8	55.0	0.0 ± 10.3	33.3
LARK	Liver	Regular motion 1	6DoF	8.8 ± 3.0	13.0	0.0 ± 5.5	16.5
			1DoF	1.6 ± 0.9	4.1	0.0 ± 1.6	5.7
LARK	Liver	Regular motion 2	6DoF	13.1 ± 6.2	24.9	0.0 ± 7.1	24.8
			1DoF	3.7 ± 2.0	8.9	0.0 ± 2.8	8.4

The 6DoF motion platform trace data is from the component of largest translational motion (superior-inferior) which was actuated in the anterior-posterior direction in the validation tests in this work. The 6DoF motion platform actuated internal patient tumour motion while the 1DoF motion platform actuated external patient surface motion

The accuracy of time synchronisation between the two motion platforms was calculated using a Root Mean Square Error (RMSE) method. The output logs from the depth-sensing camera included amplitude and time information. For each of the 6DoF and 1DoF platforms, the input and output motion traces were aligned by minimising the RMSE value of the motion amplitude. The time synchronisation accuracy was calculated as the difference between the average time values of the 6DoF and 1DoF output motion traces once aligned. For both the laboratory and clinic testing, the cross-correlation lag between the input and output motion traces was also calculated as the time delay between the input and measured output motions which led to the highest cross correlation coefficients. Additional metrics calculated for laboratory testing included the per-trace RMSE and repeatability coefficient across the repeated runs, Bland-Altman 95% limits of agreement, and windowed RMSE between input and measured output motions across consecutive non-overlapping 10-second windows.

For the system testing in the radiotherapy clinic, the 6DoF motion platform’s performance was verified by comparing the input traces with the output from the 6DoF motion platform’s positional feedback system (see Section “IntERAct motion control software”). The 1DoF motion platform’s performance was verified with the depth-sensing camera. Four motion traces were tested in the clinic for a test of the geometric accuracy and repeatability of the 6DoF and 1DoF platforms. Per-trace RMSE values, Bland-Altman 95% limits of agreement, and windowed RMSE across consecutive non-overlapping 10-second windows were also calculated for the validation tests in the clinic.

A power-spectral density comparison between input and measured motion was also calculated for the 6DoF and 1DoF platforms to measure fidelity in the respiratory frequency band using Welch’s power spectral density estimate for the validation tests in the laboratory and the clinic. The power spectral density (PSD) describes how the power is distributed across the frequency components of a signal. For a signal $x\left(t\right)$, the PSD ${P}_{xx}\left(f\right)$ is defined in Eq. (2) as:

2 $${P}_{xx}\left(f\right)=\lim\limits_{T\to \infty}\frac{1}{T}{\left|{X}_{T}\left(f\right)\right|}^{2}$$

where ${X}_{T}\left(f\right)$ is the Fourier transform of the signal over time interval $T$. Welch’s method involves segmenting the signal into overlapping windows in the time domain and computing the periodogram of each segment, then averaging the periodograms. The resulting PSD output ${P}_{xx}\left(f\right)$ is the estimate in units of power per frequency (mm^2^/Hz here). This result was integrated over the respiratory frequency band to obtain the power in mm^2^ for the input and measured output signals, and the power ratio calculated as the ratio of the input to the output power. The normal rate of respiration in healthy adults is 12–20 breaths per minute (0.2–0.33 Hz) [35]. Some components of the respiratory signal could be slightly outside this range, so the tested respiratory frequency band was 0.15–0.4 Hz when calculating the power ratio.

**Fig. 4:**
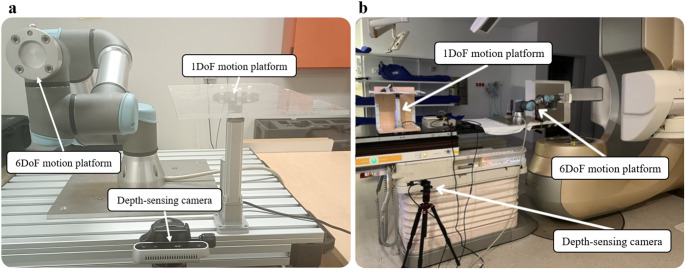
**a** IntERAct measurement setup in the laboratory. **b** IntERAct measurement setup in the radiotherapy clinic

Figure 4a illustrates the IntERAct laboratory measurement setup while Fig. 4b shows the clinic measurement setup.

## Results

**Fig. 5:**
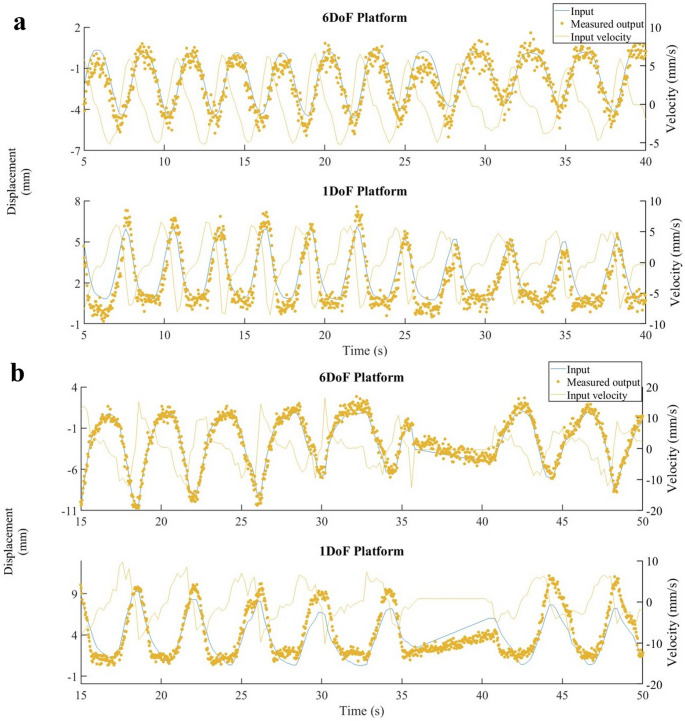
**a** Lung Regular Motion 1 input versus output motion traces measured by a depth-sensing camera in the AP direction of the 6DoF and 1DoF motion platforms in the laboratory. **b** Liver Regular Motion 1 input versus output motion traces in the AP direction of the 6DoF and 1DoF motion platforms in the laboratory. For **a** and **b** the input motion velocities are also overlaid

**Fig. 6:**
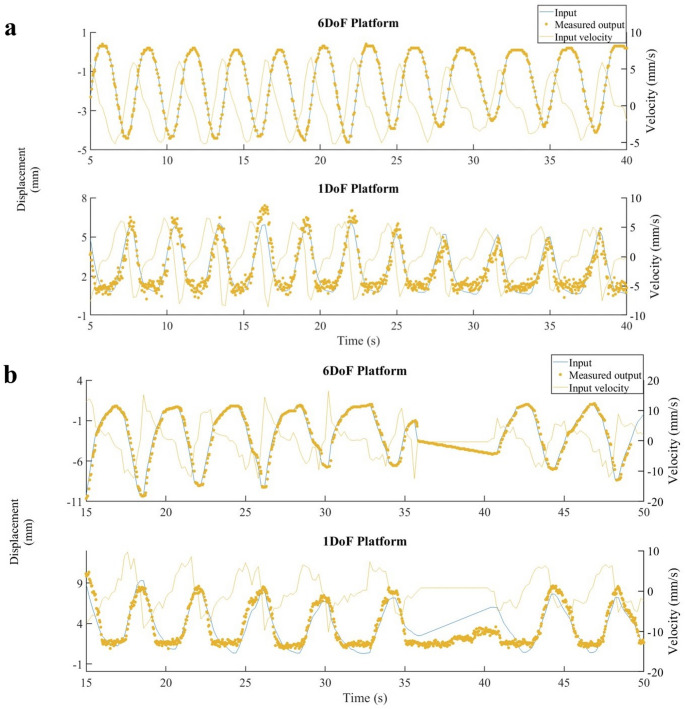
**a** Lung Regular Motion 1 input versus output motion traces for 6DoF and 1DoF motion platforms in the clinic. **b** Liver Regular Motion 1 input versus output motion traces for 6DoF and 1DoF motion platforms in the clinic. For both **a** and **b** the input motion velocities are also overlaid

The validation tests of the IntERAct QA device were performed in laboratory and radiotherapy clinic settings. Figures 5 and 6 show two example traces that have been replicated by IntERAct, in the laboratory and clinic respectively.

Tables 2 and 3 give a summary of the accuracy of the IntERAct device against the ground truth input data for all traces tested in the laboratory and the clinic respectively. In the laboratory testing, both the 6DoF and 1DoF motion platforms demonstrate repeatable millimetric accuracy (mean ± standard deviation ≤ 0.0 mm ± 1.9 mm across all traces). For the testing in the clinic, the mean errors as measured by the 6DoF platform’s feedback system were consistently ≤ 0.0 mm with the standard deviation ≤ 0.1 mm and RMSE ≤ 0.1 mm. The mean errors of the 1DoF platform as tested in the clinic were repeatably ≤ 0.0 mm with a standard deviation and RMSE of ≤ 1.3 mm over the four regular motion traces. The average time synchronisation error between the platforms was 0.1 s ± 0.0 s as measured in the laboratory across three trials. The measured cross-correlation lag between input and output motion traces was < 0.1 s for all laboratory and clinic measurements.

**Table 2:** Differences between input and output motion for 7 motion traces, measured using a depth-sensing camera for the 6DoF and 1DoF platform components of the IntERAct QA device as tested in the laboratory

Motion trace	Platform	Mean error ± standard deviation (mm)	RMSE (mm)	1st percentile of error (mm)	99th percentile of error (mm)	Bland–Altman 95% limits of agreement (mm)	Repeatability coefficient (mm)
LIGHT SABR—Lung regular motion 1	6DoF	− 0.0 ± 0.6	0.6	− 1.5	1.4	[− 1.2, 1.2]	1.7
	1DoF	0.0 ± 0.8	0.8	− 1.8	2.0	[− 1.5, 1.5]	2.1
LIGHT SABR—Lung regular motion 2	6DoF	− 0.0 ± 0.6	0.6	− 1.4	1.4	[− 1.2, 1.2]	1.7
	1DoF	0.0 ± 1.0	1.0	− 2.4	2.4	[− 1.9, 1.9]	2.7
LIGHT SABR—Lung high motion	6DoF	− 0.0 ± 0.8	0.8	− 1.9	1.7	[− 1.5, 1.5]	2.1
	1DoF	0.0 ± 1.2	1.2	− 2.8	3.3	[− 2.4, 2.4]	3.4
LIGHT SABR—Lung regular complexity	6DoF	0.0 ± 0.7	0.7	− 1.7	1.7	[− 1.4, 1.4]	2.0
	1DoF	0.0 ± 1.1	1.1	− 2.6	2.9	[− 2.2, 2.2]	3.1
LIGHT SABR—Lung high complexity	6DoF	− 0.0 ± 0.7	0.7	− 1.7	1.6	[− 1.4, 1.4]	2.0
	1DoF	− 0.0 ± 1.9	1.9	− 4.7	4.4	[− 3.7, 3.7]	5.3
LARK—Liver regular motion 1	6DoF	− 0.0 ± 0.6	0.6	− 1.4	1.4	[− 1.2, 1.2]	1.7
	1DoF	0.0 ± 1.1	1.1	− 2.5	2.9	[− 2.2, 2.2]	3.1
LARK—Liver regular motion 2	6DoF	− 0.0 ± 0.6	0.6	− 1.5	1.5	[− 1.2, 1.2]	1.8
	1DoF	0.0 ± 1.1	1.1	− 2.4	2.6	[− 2.1, 2.1]	2.9

**Table 3:** Differences between input and output motion for 4 motion traces, measured using the 6DoF robotic feedback system (6DoF platform) and the depth sensing camera for the 1DoF platform of the IntERAct QA device in the clinic

Motion trace	Platform	Mean error ± standard deviation (mm)	RMSE (mm)	1st percentile of error (mm)	99th percentile of error (mm)	Bland–Altman 95% limits of agreement (mm)
LIGHT SABR – Lung Regular Motion 1	6DoF	− 0.0 ± 0.1	0.1	− 0.2	0.2	[− 0.2, 0.1]
	1DoF	0.0 ± 0.7	0.7	− 3.2	1.7	[− 1.3, 1.3]
LIGHT SABR – Lung Regular Motion 2	6DoF	− 0.0 ± 0.1	0.1	− 0.3	0.2	[− 0.2, 0.2]
	1DoF	0.0 ± 0.8	0.8	− 1.8	1.7	[− 1.5, 1.5]
LARK – Liver Regular Motion 1	6DoF	0.0 ± 0.1	0.1	− 0.4	0.4	[− 0.3, 0.3]
	1DoF	− 0.0 ± 1.3	1.3	− 3.3	2.3	[− 2.5, 2.5]
LARK – Liver Regular Motion 2	6DoF	0.0 ± 0.1	0.1	− 0.3	0.4	[− 0.3, 0.3]
	1DoF	0.0 ± 1.0	1.0	− 1.8	3.0	[− 2.0, 2.0]

Plots of the windowed RMSE results across consecutive non-overlapping 10 s windows for all traces in the laboratory and clinic testing are presented in the supplementary material. The measured windowed RMSE for the 1DoF platform increased by up to 0.9–1 mm for time windows covering sections of the motion traces which incorporated linear motion, as in the time segment ~ 30–40 s in the LARK Liver Regular motion 1 trace presented in Fig. 6b.

A power-spectral density comparison between input and measured motion was conducted with plots and the power ratio details added to the supplementary material. The power ratio of the input and measured motions for the 6DoF platform in the respiratory frequency band (0.15–0.4 Hz) ranged from 0.99 to 1.07 (mean ± standard deviation: 1.02 ± 0.03) for the laboratory testing and from 1.00 to 1.01 (mean ± standard deviation: 1.00 ± 0.01) for the clinical testing. The power ratio of the input and measured motions for the 1DoF platform in the respiratory frequency band ranged from 0.96 to 1.55 (mean ± standard deviation: 1.23 ± 0.23) for the laboratory testing and from 0.69 to 1.01 (mean ± standard deviation: 0.78 ± 0.15) for the clinical testing.

The source code, phantom design, user guides for the system, and instructions to perform QA tests with the IntERAct device have been compiled in an open-source repository on GitHub [29] for other research groups to use and modify. The IntERAct platform’s motion control software used for the experimental validation in this work is located on a publicly accessible GitHub repository: https://github.com/Image-X-Institute/IntERAct/releases/download/v1.1.0-alpha/IntERAct-Release-v1.1.3-alpha.zip.

## Discussion

In this work, we developed and characterised the performance of IntERAct, an open-source QA device that reproduces synchronised motion on a 6 degrees-of-freedom robotic motion platform and 1 degree-of-freedom motion platform. The IntERAct QA device was characterised in both laboratory and clinical settings to simulate time-synchronised internal and external motion. The QA device will support the quality assurance of real-time IGRT technologies that rely on internal and external motion measurements to estimate target position or multi-target tracking technologies. The addition of a low-cost 1DoF actuator ensures that the combined system is easily transportable and is less expensive than creating a combined system of two 6DoF robots.

The IntERAct device demonstrates capabilities of highly repeatable motion simulation as measured by an independent system (RealSense depth-sensing camera). Seven motion traces tested in laboratory and radiotherapy clinic settings demonstrated consistent and repeatable accuracy and precision (mean errors all ≤ 0.0 mm and standard deviation all ≤ 1.9 mm), with time synchronisation between the two platforms of 0.1 ± 0.0s. The cross-correlation lag between input and measured motion for the 6DoF and 1DoF platforms respectively was < 0.1 s for all measured traces supporting the accurate time response characteristics of the two component platforms. The LIGHT SABR—Lung High Complexity motion trace achieved a favourable mean error of 0.0 mm over the three laboratory tests in the 1DoF platform component, but had a standard deviation of 1.9 mm. However, this input motion trace demonstrated a 1DoF external motion amplitude reaching 60 mm, which is rarely encountered [36]. From the results across the laboratory and the clinic, the IntERAct QA device mean positional error is ≤ 0.0 mm and RMSE is ≤ 0.8 mm for the 6DoF platform component and ≤ 0.0 mm (mean positional error) and ≤ 1.9 mm (RMSE) for the 1DoF platform component. This is within the recommended AAPM spatial accuracy threshold for medical linear accelerator QA of 2 mm [30].

We note that the RealSense camera measurements have not been directly compared against the 6DoF motion platform’s positional feedback in the laboratory testing. However, the 6DoF motion platform’s positional accuracy has been extensively validated in previous work [18] to < 0.1 mm mean positional error and < 0.2 mm RMSE in each translational direction. The generally comparable results of the measured accuracy of the 6DoF platform in the laboratory testing (mean positional error of 0.0 mm and mean RMSE of 0.7 mm) support the use of the RealSense measurements as a measure of the positional accuracy of the IntERAct system to within 1 mm. To further improve the positional accuracy of the 1DoF motion platform component, the linear actuator could be changed to a more accurate motor, with the limitation that this component would be more expensive [37]. Alternatively, another 6DoF arm could be used as a second platform component, depending on the usage requirements of the system.

For the laboratory testing, the repeatability coefficient across the three repeated runs was reported in Table 2 as a measure of the maximum expected difference between three repeated measurements of the platform’s motion under the same conditions. The standard deviation did not differ by more than 0.1 mm for the 6DoF platform and 0.2 mm for the 1DoF platform traces across the repeated tests, demonstrating high repeatability, so the largest standard deviation across the three tests is likely to be a conservative measure of the repeatability of the platform operation. The largest standard deviation measured in all tests of the 6DoF platform was 0.8 mm and this result was 1.9 mm for the 1DoF platform.

The windowed RMSE results (across 10 s windows) for the 1DoF platform demonstrated lower accuracy (up to 0.9–1 mm higher RMSE) in windows containing artificial linear motion. This linear motion was not physically realistic and was introduced to the motion traces to replace sections of the original motion traces that exhibited significant outliers due to marker segmentation errors. The linear motion was introduced to prevent discontinuities in the motion signal. It is possible that the RealSense camera or 1DoF platform performance was impaired in these regions of slow (< 2 mm/s) linear motion of the planar 1DoF platform surfaces, in contrast to its acceptable performance in measuring the respiratory motions and it is recommended to minimise the presence of physiologically unrealistic motions in the input motion traces when operating the platform.

A power-spectral density comparison for the 6DoF and 1DoF platform components was conducted with results presented in supplementary material. Power ratio values around 1 suggest high fidelity of the motion actuation. While the 6DoF platform exhibited an acceptable power ratio of 1.0-1.1 for all tests, the 1DoF platform exhibited slightly less reproducible behaviour. In the laboratory testing the 1DoF platform exhibited moderate (~ 20%) noise or amplification of these frequencies (mean power ratio 1.2), whereas in the clinical testing there was moderate (~ 20%) signal attenuation (mean power ratio 0.8). These results are likely to be expected for a low-cost actuator, and as the geometric accuracy characterisation has been found to show acceptable results, the performance of the 1DoF motion platform was considered sufficient for our purposes.

The IntERAct device offers comparable translational accuracy to other motion platforms, such as the 4DoF system by Mukumoto et al.. (2016), which demonstrated a mean positional error of 0.1 mm [38]. Similarly, the accuracy of the 6DoF motion platform is comparable to the commercially available 5DoF HexaMotion system, which demonstrated a mean positional error of reproducing dynamic patient motion of ≤ 0.3 mm [14]. IntERAct offers the advantage of an additional degree of freedom and a significantly larger range of rotation of 360° around each rotational axis, while integrating a 1DoF platform that replicates the external signal synchronously with the robotic arm. This can enable full representation of clinically observed motion of up to 45° in lung [15] and 29° in liver [16]. The IntERAct device’s dual-motion capability will be especially beneficial in QA of IGRT technologies treating tumours in the thorax and abdomen that utilise the correlation between internal tumour and external surface motion for motion monitoring [11, 39].

A key advantage of the IntERAct QA device is its enhanced capacity to support heavier phantoms (up to 16 kg), enabling use of more realistic phantoms and the integration of dosimeters [22] for a broader range of measurements, including those required for safe clinical implementation of marker-less real-time IGRT technologies [21]. This device will be prospectively used as part of *A Master Trial Assessing the Technical Feasibility of First-In-Human Real-Time Image Guided Radiation Therapy Methods* (Real-Time IGRT) master trial (NCT06708221).

Incorporating a low-cost linear actuator for the surface motion actuation makes the system inexpensive, lightweight, and portable for multi-site use. A current limitation is that the device’s external motion replication is only in 1DoF, while surface motion is known to have multiple motion components. Future directions include integrating platforms capable of replicating 3DoF or 6DoF external signals to better represent complex physiological motion. Tumours and surrounding tissues also often deform during treatment due to factors such as pressure from adjacent organs or changes in the patient’s position [17]. The platform can be further developed and validated for scenarios where tumour volume varies during the treatment session, by incorporating deformable phantom components, which could also expand applications to surface-guided radiotherapy quality assurance [17]. Additionally, tumours located near the heart or in regions affected by complex cardio-respiratory motion pose a challenge for radiotherapy [40]. Future work will develop the IntERAct device’s functionality to replicate complex cardio-respiratory motion, thereby increasing its applicability to quality assurance of IGRT technologies for cancer and cardiac diseases [41].

The source code, phantom design, user guides to integrate the system and instructions to perform QA tests with the IntERAct device have been compiled in an open-source repository on GitHub for other research groups to use, modify and redistribute [29]. This device has been used in three multi-institutional clinical trials in 12 radiation therapy departments in Australia enabling IGRT QA and routine linear accelerator QA. Future developments will be updated regularly and made accessible to the medical physics community.

## Conclusion

IntERAct, the synchronised 6DoF and 1DoF motion platform developed in this work, is a novel quality assurance device for motion management in image-guided radiotherapy. By integrating two independent motion platforms into a single, synchronised system, this platform helps to address a challenging aspect of radiotherapy - accurately targeting tumours that move due to physiological activities such as respiration. The system exhibits sub-two-millimetre accuracy with high repeatability, enabling the replication of complex 6DoF tumour motion in addition to 1DoF surface motion, facilitating enhanced radiotherapy precision for treatment sites that move with respiration. The IntERAct system will be used to experimentally validate and conduct QA of real-time combined internal-external motion monitoring technologies. The code, phantom design, and hardware specifications are open-source for the medical physics community to use and edit for other QA purposes.

Statements and Declarations.

## References

[1] Keall PJ et al (2006) The management of respiratory motion in radiation oncology report of AAPM Task Group 76a. Med Phys 33(10), 3874–3900. 10.1118/1.2349696 10.1118/1.2349696 17089851

[2] Bertholet J et al (2019) Real-time intrafraction motion monitoring in external beam radiotherapy. Phys Med Biol 64(15):15TR01. 10.1088/1361-6560/ab2ba8 10.1088/1361-6560/ab2ba8 PMC7655120 31226704

[3] Seppenwoolde Y, Berbeco RI, Nishioka S, Shirato H, Heijmen B (2007) Accuracy of tumor motion compensation algorithm from a robotic respiratory tracking system: a simulation study. Med Phys 34(7):2774–2784. 10.1118/1.2739811 10.1118/1.2739811 17821984

[4] Ferris WS, Kissick MW, Bayouth JE, Culberson WS, Smilowitz JB (2020) Evaluation of radixact motion synchrony for 3D respiratory motion: Modeling accuracy and dosimetric fidelity. J Appl Clin Med Phys 21(9):96–106. 10.1002/acm2.12978 10.1002/acm2.12978 PMC7497925 32691973

[5] Jin J-Y, Yin F-F, Tenn SE, Medin PM, Solberg TD (2008) Use of the brainlab exactrac X-ray 6D system in image-guided radiotherapy. Med Dosim 33(2):124–134. 10.1016/j.meddos.2008.02.005 18456164 10.1016/j.meddos.2008.02.005

[6] Keall PJ, Barton M, Crozier S (2014) The Australian magnetic resonance imaging–linac program. Semin Radiat Oncol 24(3):203–206. 10.1016/j.semradonc.2014.02.015 10.1016/j.semradonc.2014.02.015 24931094

[7] Ozhasoglu C et al (2008) Synchrony—cyberknife respiratory compensation technology. Med Dosim 33(2):117–123. 10.1016/j.meddos.2008.02.004 18456163 10.1016/j.meddos.2008.02.004

[8] Yang B et al (2021) Comparison of modeling accuracy between radixact^®^ and cyberKnife^®^ synchrony^®^ respiratory tracking system. Biomed Phys Eng Express 7(6):67001. 10.1088/2057-1976/ac1fa5 10.1088/2057-1976/ac1fa5 34416743

[9] Bertholet J et al (2018) Automatic online and real-time tumour motion monitoring during stereotactic liver treatments on a conventional Linac by combined optical and sparse monoscopic imaging with kilovoltage x-rays (COSMIK). Phys Med Biol 63(5):55012. 10.1088/1361-6560/aaae8b 10.1088/1361-6560/aaae8b 29516868

[10] Skouboe S et al (2019) First clinical real-time motion-including tumor dose reconstruction during radiotherapy delivery. Radiother Oncol 139:66–71. 10.1016/j.radonc.2019.07.007 10.1016/j.radonc.2019.07.007 31431367

[11] Nguyen DT et al (2018) An augmented correlation framework for the estimation of tumour translational and rotational motion during external beam radiotherapy treatments using intermittent monoscopic x-ray imaging and an external respiratory signal. Phys Med Biol 63(20):205003. 10.1088/1361-6560/aadf2c 30183677 10.1088/1361-6560/aadf2c

[12] Tohyama N, Saito E, Uchida K, Yoda K, Mori S (2024) The Itappachi universal motion platform for accurate dose measurement in thoracoabdominal radiotherapy. Cureus 10(16):e71713. 10.7759/cureus.71713 10.7759/cureus.71713 PMC11568831 39553058

[13] Saito M et al (2023) Development of a dynamic motion platform with two independent drive systems for radiotherapy. J Appl Clin Med Phys 24(5):e13971. 10.1002/acm2.13971 10.1002/acm2.13971 PMC10161022 36951306

[14] Huang C-Y, Keall P, Rice A, Colvill E, Ng JA, Booth JT (2017) Performance assessment of a programmable five degrees-of-freedom motion platform for quality assurance of motion management techniques in radiotherapy. Australas Phys Eng Sci Med 40(3):643–649. 10.1007/s13246-017-0572-0 28717901 10.1007/s13246-017-0572-0

[15] Plathow C et al (2006) Quantification of lung tumor volume and rotation at 3D dynamic parallel MR imaging with view sharing: preliminary results. Radiology 240(2):537–545. 10.1148/radiol.2401050727 16801367 10.1148/radiol.2401050727

[16] Bertholet J, Worm ES, Fledelius W, Høyer M, Poulsen PR (2016) Time-resolved intrafraction target translations and rotations during stereotactic liver radiation therapy: implications for marker-based localization accuracy. Int J Radiat Oncol Biol Phys 95(2):802–809. 10.1016/j.ijrobp.2016.01.033 27020108 10.1016/j.ijrobp.2016.01.033

[17] Cheung Y, Sawant A (2015) An externally and internally deformable, programmable lung motion phantom. Med Phys 42(5):2585–2593. 10.1118/1.4918581 25979050 10.1118/1.4918581 PMC4409628

[18] Alnaghy S et al (2019) A six-degree-of-freedom robotic motion system for quality assurance of real-time image-guided radiotherapy. Phys Med Biol 64(10):105021. 10.1088/1361-6560/ab1935 30986773 10.1088/1361-6560/ab1935

[19] UR3 CB 3 Ultra-lightweight compact industrial collaborative robot arm, Universal Robots. Accessed 22 April 2025. Available: https://www.universal-robots.com/media/1828034/ur3_tech_spec_web_en.pdf

[20] UR16e Heavy-duty, compact collaborative robot, Universal Robots. Accessed 22 April 2025. Available: https://www.universal-robots.com/products/ur16-robot/

[21] Mueller M et al (2020) The first prospective implementation of markerless lung target tracking in an experimental quality assurance procedure on a standard linear accelerator. Phys Med Biol 65(2):025008. 10.1088/1361-6560/ab5d8b 10.1088/1361-6560/ab5d8b 31783395

[22] Hindmarsh J et al (2025) A dosimetric comparison of helical tomotherapy treatment delivery with real-time adaption and no motion correction. Phys Imaging Radiat Oncol 34:100741. 10.1016/j.phro.2025.100741 10.1016/j.phro.2025.100741 PMC11931245 40129726

[23] Universal Robots UR3 User Manual,, Universal Robots AS, Accessed 09 October 2025. Available: https://s3-eu-west-1.amazonaws.com/ur-support-site/21984/UR3_User_Manual_en_Global.pdf

[24] Optical Feedback Linear Actuators Firgelli Automations Australia. Accessed 08 April 2025. Available: https://www.firgelliauto.com.au/products/os-series

[25] Double DC, Motor Driver PWM, Phipps Electronics. Accessed: 22 April 2025. Available: https://www.phippselectronics.com/product/bts7960-43a-double-dc-stepper-motor-driver-h-bridge-pwm/?gad_source=1&gclid=EAIaIQobChMIh4e8mK2OhgMVbKtmAh3BWwXsEAQYASABEgJUQfD_BwE

[26] Arduino, UNO R3 specifications, Arduino. Accessed 22 April 2025. Available: https://docs.arduino.cc/hardware/uno-rev3/#tech-specs

[27] Intel RealSense Documentation Intel RealSense. Accessed 22 April 2025. Available: https://dev.intelrealsense.com/docs/docs-get-started

[28] Arduino Serial Communication Arduino. Accessed 22 April 2025. Available: https://www.arduino.cc/reference/en/language/functions/communication/serial/

[29] Alnaghy S et al IntERAct, GitHub. Accessed 22 April 2025. Available: https://github.com/Image-X-Institute/IntERAct

[30] Kutcher GJ et al (1994) Comprehensive QA for radiation oncology: report of AAPM radiation therapy committee task group 40. Med Phys 21(4):581–618. 10.1118/1.597316 8058027 10.1118/1.597316

[31] Klein EE et al (2009) Task Group 142 report: quality assurance of medical acceleratorsa. Med Phys 36(9Part1):4197–4212. 10.1118/1.3190392 10.1118/1.3190392 19810494

[32] Booth JT et al (2016) The first patient treatment of electromagnetic-guided real time adaptive radiotherapy using MLC tracking for lung SABR. Radiother Oncol 121(1):19–25. 10.1016/j.radonc.2016.08.025 27650013 10.1016/j.radonc.2016.08.025

[33] Sengupta C et al (2024) The first clinical implementation of real-time 6 degree-of-freedom image-guided radiotherapy for liver SABR patients. Radiother Oncol 190:110031. 10.1016/j.radonc.2023.110031 38008417 10.1016/j.radonc.2023.110031

[34] Boussuges A, Finance J, Chaumet G, Brégeon F (2021) Diaphragmatic motion recorded by M-mode ultrasonography: limits of normality. ERJ Open Res 7(1):00714–02020. 10.1183/23120541.00714-2020 10.1183/23120541.00714-2020 PMC7983192 33778044

[35] Nicholson TW, Talbot NP, Talbot NP, Nickol A, Chadwick AJ, Lawton O (2020) Respiratory failure and non-invasive respiratory support during the COVID-19 pandemic: an update for re-deployed hospital Doctors and primary care physicians. BMJ 10.1136/bmj.m2446 10.1136/bmj.m2446 32605992

[36] Wang G et al (2022) Correlation of optical surface respiratory motion signal and internal lung and liver tumor motion: a retrospective Single-Center observational study. Technol Cancer Res Treat 21:15330338221112280. 10.1177/15330338221112280 10.1177/15330338221112280 PMC9272160 35791642

[37] Schneider B et al Design, manufacturing, and testing of precision space flight qualified single degree of freedom flexure based linear actuators/mechanisms.

[38] Mukumoto N et al (2016) Development of a four-axis moving phantom for patient‐specific QA of surrogate signal‐based tracking IMRT. Med Phys 43(12):6364–6374. 10.1118/1.4966130 27908156 10.1118/1.4966130 PMC5648581

[39] Kaczynska A et al (2024) First experimental investigation of a real-time 6 degrees-of-freedom tumor motion monitoring device for thoracic and abdominal cancer sites. In: AAPM 66th annual meeting and exhibition, AAPM. Accessed 08 April 2025. Available: https://aapm.confex.com/aapm/2024am/meetingapp.cgi/Paper/11056

[40] Stevens RRF et al (2024) A framework for assessing the effect of cardiac and respiratory motion for stereotactic arrhythmia radioablation using a digital phantom with a 17-segment model: A STOPSTORM.eu consortium study. Int J Radiat Oncol Biol Phys 118(2):533–542. 10.1016/j.ijrobp.2023.08.059 37652302 10.1016/j.ijrobp.2023.08.059

[41] Lydiard S, Blanck O, Hugo G, O’Brien R, Keall P (2021) A review of cardiac radioablation (CR) for arrhythmias: procedures, technology, and future opportunities, Elsevier Inc. 10.1016/j.ijrobp.2020.10.036 10.1016/j.ijrobp.2020.10.036 33160007

